# Towards a better understanding of anticipatory postural adjustments in people with Parkinson’s disease

**DOI:** 10.1371/journal.pone.0300465

**Published:** 2024-03-11

**Authors:** Jana Seuthe, Anna Heinzel, Femke Hulzinga, Pieter Ginis, Kirsten E. Zeuner, Günther Deuschl, Nicholas D’Cruz, Alice Nieuwboer, Christian Schlenstedt

**Affiliations:** 1 Institute of Interdisciplinary Exercise Science and Sports Medicine, MSH Medical School Hamburg, Hamburg, Germany; 2 Department of Neurology, University Hospital Schleswig-Holstein, Kiel, Germany; 3 Department of Rehabilitation Sciences, KU Leuven, Leuven, Belgium; Aston University, UNITED KINGDOM

## Abstract

**Introduction:**

Previous studies have shown that anticipatory postural adjustments (APAs) are altered in people with Parkinson’s disease but its meaning for locomotion is less understood. This study aims to investigate the association between APAs and gait initiation, gait and freezing of gait and how a dynamic postural control challenging training may induce changes in these features.

**Methods:**

Gait initiation was quantified using wearable sensors and subsequent straight walking was assessed via marker-based motion capture. Additionally, turning and FOG-related outcomes were measured with wearable sensors. Assessments were conducted one week before (Pre), one week after (Post) and 4 weeks after (Follow-up) completion of a training intervention (split-belt treadmill training or regular treadmill training), under single task and dual task (DT) conditions. Statistical analysis included a linear mixed model for training effects and correlation analysis between APAs and the other outcomes for Pre and Post-Pre delta.

**Results:**

52 participants with Parkinson’s disease (22 freezers) were assessed. We found that APA size in the medio-lateral direction during DT was positively associated with gait speed (p<0.001) and stride length (p<0.001) under DT conditions at Pre. The training effect was largest for first step range of motion and was similar for both training modes. For the associations between changes after the training (pooled sample) medio-lateral APA size showed a significant positive correlation with first step range of motion (p = 0.033) only in the DT condition and for the non-freezers only.

**Conclusions:**

The findings of this work revealed new insights into how APAs were not associated with first step characteristics and freezing and only baseline APAs during DT were related with DT gait characteristics. Training-induced changes in the size of APAs were related to training benefits in the first step ROM only in non-freezers. Based on the presented results increasing APA size through interventions might not be the ideal target for overall improvement of locomotion.

## Introduction

Start hesitation in Parkinson’s disease (PD) is a disturbance of the gait initiation (GI) process resulting in impaired mobility and a loss of independence. Studying GI in PD therefore has been of particular interest to develop effective treatment [1]. The GI process contains the preparation- and the execution phase: The preparation phase is the anticipatory postural adjustment (APA) which is usually characterized by two components: a shift of the center of pressure from anterior to posterior (AP) and by a medio-lateral (ML) shift towards the swing foot to accelerate the center of mass anteriorly towards the stance foot [2,3]. The execution phase includes the size and timing of the first step. Through the APA the center of mass is prevented from falling towards the swing foot, which increases stability during the single leg stance phase of the first step [4], however a more lateral placement of the swing foot (increasing step width) can also attain this effect [5]. While studies exist having investigated various measures of the GI process for people with PD itself, little is known about the associations and the role of measures of GI and locomotion.

Interestingly, it has been observed that GI can be performed without an adequate APA but by the postural sway during stance prior gait [6,7], yielding the question about the role of APAs for adequate GI and the subsequent gait quality. Findings on possible associations between APA outcomes and first step metrics or the following steady state gait are scarce. Brenière et al. [8] showed that steady state gait velocity in healthy individuals is significantly correlated with the size and duration of the AP component of APAs, indicating that larger backward APAs are associated with faster gait speed. Mancini et al. [9] found similar results in people with PD, showing a significant correlation between both AP and ML APA magnitudes and the first step velocity. Additionally, compared to healthy controls, individuals with PD have smaller [9–11] and longer-lasting [11] APAs. One study showed that there were no differences in GI (ML-APA size, AP-APA size and time until the first step) between people with PD and HC when outcomes were normalized to gait speed [2]. With regard to the amount of APAs before successful GI, people with PD often performed multiple or no APAs compared to healthy individuals [12]. In people with Freezing of Gait (FOG) GI can be particularly disrupted as this symptom is characterized by motor blocks and the inability to start walking [13]. As for individuals with FOG ML size of APA was smaller than in PD without FOG when an additional cognitive task was performed simultaneously [14]. Comparing the successful and unsuccessful reinitiation of gait (after a freeze) in PD with FOG has shown that successful trials have a larger preceding ML weigh-shift [15]. However, controversially another study reported that people with higher self-reported freezing severity have larger ML-APAs [14]. The authors speculated that smaller ML-APAs in those without FOG might be a compensatory strategy enabling successful stepping during GI.

Regarding the role of dual tasking during GI the literature is not consistent. While one study found differences between the single- and dual task conditions [14], other studies did not find an effect of dual tasking [16,17].

Gait impairment including disrupted GI can have a negative impact on quality of life in people with PD [18] and is associated with falls [19]. However, few studies investigated possible therapeutic effects to improve GI. Levodopa therapy can alleviate the previously described APA disturbances to some extent by increasing force production during GI as well as APA amplitude [10,20], but not in all patients and especially not in those with FOG [21]. Furthermore, there is a limited number of studies investigating the potential of training interventions to modulate GI in people with PD. Two studies showed that APA size could be significantly increased, using progressive resistance training [22] and Qi-Gong [23]. Additionally, an agility boot camp program improved first step length [24]. However, these studies did not explore whether changes in APAs related to changes in GI, gait and FOG-related measures. Despite being a well-researched exercise form for gait rehabilitation in PD to the best of our knowledge treadmill training has not been studied in the context of modulating GI. Regular treadmill training is expected to improve overall gait outcomes like gait speed or stride length, which could consequently lead to a larger first step during GI as well. Recently split-belt-treadmill (SBT) training has gained more interest as a possible rehabilitation option for people with PD. A SBT consists of two parallel belts, one for each leg, whereby the speeds can be controlled separately, enabling the possibility to introduce asymmetric perturbations and dynamic walking conditions. Walking with changing belt speeds requires adapted weight shifts of the body’s center of mass, challenging dynamic postural control. SBT training can thus be expected to enhance the controlled shift of the center of mass and by implication potentially influence GI more than traditional treadmill training.

Due to the described gap in the current literature, this study aims at investigating the role of APAs for locomotion in individuals with PD with and without FOG. First, we investigated whether APAs were related to measures of locomotion by correlating APAs with first step metrics, overground gait, and FOG-related outcomes at baseline. Second, we investigated whether SBT training affected APA outcomes more than regular treadmill training and whether training benefits could be linked with changes in GI, overground gait and FOG-related measures.

## Methods

This is a sub-analysis of a randomized-controlled trial investigating the effects of SBT training compared to regular treadmill training on gait adaptability (Clinical Trial No: NCT04176263) [25]. Fifty-two individuals with PD (22 with FOG) were recruited for this study between 12^th^ August 2019 and 24^th^ February 2021. Eligibility criteria were the diagnosis of idiopathic Parkinson’s disease, Hoehn and Yahr stage 2–3, the ability to walk independently for at least five minutes, absence of other neurological disorders, absence of orthopedic or other conditions that influences gait or balance, and absence of severe cognitive impairment (Mini Mental State examination <24). Patients were classified as freezers when they had 1 point for item 1 of the NFOG-Q or a trained assessor visually detected FOG during a FOG-provoking turning in place task, whereas all other participants were classified as non-freezers. Participants were randomized into either the SBT training or regular treadmill training group upon inclusion based on their freezer classification (freezer/non-freezer) and Hoehn and Yahr stage (2 or 3). The training protocol of this study has been described in detail elsewhere [25]. In brief, participants trained 3 times per week for 4 weeks either receiving different SBT contrasts with increasing difficulty (more switches with larger contrasts) or systematic increases of the treadmill speed for the regular treadmill training. In addition, in both groups duration of the sessions was gradually increased from 30 minutes to 45 minutes. Assessments were carried out the week before (Pre), the week after (Post) and four weeks after the training intervention (Follow-up). Assessments and training sessions were conducted in the ON medication state. The local ethics committees approved the study (CAU Kiel: D 454/13; KU Leuven: S62825) and participants gave written informed consent prior to participation.

### Assessment protocol

Assessment of APAs and first step outcomes was carried out using an accelerometer-based approach (APDM, Mobility Lab). Sensors were placed on the lower back and on top of each foot to record GI at a sampling frequency of 128 Hz. Participants were asked to initiate gait from a standardized stance position determined by a foot template which was placed between the feet beforehand (10 cm distance between heels and 30-degree outward rotation). Participants were asked to stand quietly for 5 seconds. Then, a visual go-signal was displayed, and participants had been instructed to initiate gait after the go-signal was presented. Participants were not asked to initiate gait with a specific limb (e.g. dominant or more affected) or always with the same limb. Assessment of subsequent overground gait (straight walking at comfortable pace, 10 m) was carried out using 3D-motion capture systems (Kiel: Qualisys, Leuven: Vicon) with reflective markers placed on the lateral ankle, the toe and the heel of each foot at a sampling frequency of 100 Hz. The gait initiation and subsequent straight walking was repeated five times for each participant. To assess objective FOG related outcomes (% time frozen and FOG ratio) participants were asked to perform a one-minute turning in place task. This task has been validated for the assessment of FOG severity in a laboratory environment [26]. The instruction was to perform turns in alternating directions as fast and safely as possible for 1 minute. FOG parameters during the turning task were assessed via video rating and using the same accelerometers, with the sensors on the feet repositioned to the shanks for this task.

The whole assessment protocol was completed twice, once without (single task = ST) and once with an additional cognitive task (dual task = DT) in the form of an auditory Stroop test. During this test participants were presented the words “high” or “low”, spoken either in a high or low pitch (congruent or incongruent stimuli). Subjects were asked to name the pitch of the stimulus as quickly as possible. Additionally, several clinical measures were assessed: the Movement-Disorders-Society Unified Parkinson’s Disease Rating Scale part III (MDS-UPDRS III), the Montreal Cognitive Assessment (MoCA), the Frontal Assessment Battery (FAB), the Trail Making Test (TMT), the Mini Balance Evaluation Systems–Test (Mini-BESTest), the Fullerton Advanced Balance Scale, the New Freezing of Gait-Questionnaire (NFOG-Q) [27] and the FOG-Score [28].

### Data processing and statistical analysis

Data processing for GI, overground gait and turning data was carried out using customized Matlab scripts [29]. The main outcomes computed for GI were ML-APA size, AP-APA size, first step range of motion (ROM), first step time. The computation of these outcomes has been described elsewhere [9]. In short, the sensor data was filtered (Butterworth filter, cut-off 3 Hz) and APAs were detected when the trunk acceleration exceeded 3 standard deviations of the postural sway measured at quiet stance. The beginning and end of an APA was defined as the moment when trunk acceleration exceeded 1 standard deviation of the postural sway during quiet stance. The outcomes computed for steady state gait were gait speed and stride length, calculated based on the detected gait events (heel-strike and toe-off) derived from the motion capture data. The FOG-related outcome measures, which were only analyzed in the subgroup with FOG were percentage (%) time frozen during the turning task rated via video [30], the NFOG-Q (total score and GI items: item 5 and 6), the FOG-score (total score and GI items: score in the section “start walking”) [28] and the FOG ratio [26] during the turning task. The FOG ratio is the square of the total power in the 3–8 Hz band, which is the frequency of the trembling, divided by the square of the total power in the 0.5–3 Hz band, which is the frequency band of the movement, from the medio-lateral accelerations of the shanks during 360-degree turns in place to quantify FOG objectively. For all outcomes we calculated the delta (value at Post minus value at Pre) to interpret changes due to the training intervention.

Statistical analysis was implemented in R Studio [31]. Baseline differences between the participants (SBT vs. TBT and Freezers vs. Non-Freezers) were investigated using Man-Whitney-U Test for ordinal scaled variables (age, disease duration, MDS-UPDRS-III, MoCA, FES-I, FAB-Scale, Mini-BESTest, NFOG-Q, FOG-Score)), Chi-squared test for categorical variables (H&Y) and independent sample t-Tests for interval-scaled variables (LEDD, TMT, FOG ratio). The associations between APA size (ML and AP) and the other outcomes were investigated by calculating Pearson correlation coefficients (interval-scaled data) and Spearman’s rank correlation coefficients (ordinal-scaled data) with a 95% confidence interval. The absolute values of the correlation coefficients were interpreted as negligible (0.00–0.09), weak (0.10–0.39), moderate (0.40–0.69) strong (0.70–0.89) and very strong (0.90–1.00) [32]. Bonferroni correction was applied to all correlations for the domains gait initiation, gait and FOG and for ST, DT and freezers and non-freezers, respectively. To investigate whether differences in correlations between freezers and non-freezers were driven by differences in disease duration a linear regression analysis was conducted with disease duration as covariate (lm.beta package). To investigate the training effects linear mixed models (lme4-package) were calculated for the main outcomes, with training group (two levels) and time (three levels) as the independent variables, center (2 levels) as covariate with an unstructured covariance matrix. The model included a random effects term with a correlated random intercept and slope for time and subject, and a Satterthwaite approximation of the denominator degrees of freedom (model formula: variable = group*time+center + (time|subject)). Histograms and Q-Q plots were visually checked for normality of the residuals and log-transformed if not meeting assumptions and post-hoc testing was conducted with Tukey adjustment for multiple comparisons. To accommodate for missing data all randomized participants were included in the mixed linear models according to the intention-to-treat approach.

## Results

Fifty-two participants with PD were included of which 22 had FOG (SBT: n = 12; TBT: n = 10). A flow-diagram of participants flow through the study is provided within the Supplementary material (S1 Fig). Table 1A and 1B show participant characteristics for both training groups (no differences) and for freezers and non-freezers (longer disease duration in freezers). A total of n = 116 trials could not be analyzed (e.g. due to too much noise in the data) and n = 180 trials showed no APA according to our definition. Those trials were not included in the further analysis.

**Table 1:** a. Participant characteristics by training group. b. Participant characteristics by Freezing status.

	**SBT** (n = 27)	**TBT** (n = 25)	**p-value**
**Age (years)**	66.41 (7.87), 51–85	64.24 (11.49), 42–90	0.539
**Duration since Diagnosis (years)**	7.61 (5.30), 1–18	6.94 (4.36), 1–16	0.713
**H&Y (1/2/3/4)**	0/22/5/0	0/21/4/0	0.810
**MDS-UPDRS-III (0–132)**	35.07 (12.76), 9–59	31.20 (13.53), 12–69	0.190
**LEDD (mg)**	645.79 (338.31), 100–1745	563.94 (318.24), 0–1378	0.408
**Mini-BESTest (0–28)**	23.93 (3.12), 17–28	22.40 (4.66), 9–27	0.243
**FAB-Scale (0–40)**	33.15 (5.17), 17–39	30.44 (7.45), 14–40	0.295
**MoCA (0–30)**	26.41 (2.39), 20–30	25.56 (2.63), 20–29	0.304
**TMT-A (s)**	42.39 (14.70), 21.74–85	48.95 (43.71), 23.31–239	0.481
**TMT-B (s)**	92.46 (46.94), 35–213	122.66 (109.22), 44.37–582	0.089
**FES-I (16–64)**	25.63 (9.56), 16–47	24.13 (8.72), 16–50	0.629
**NFOG-Q (1–28)**	14.33 (5.41), 6–23	16.30 (5.87), 6–26	0.485
**FOG-Score (0–36)**	1.26 (2.60), 0–10	3.08 (5.06), 0–18	0.148
**FOG ratio**	3.24 (3.77), 0.44–19.18	2.99 (3.01), 0.67–14.48	0.796
	**Non-Freezers** (n = 30)	**Freezers** (n = 22)	**p-value**
**Age (years)**	65.10 (10.78), 42–90	65.73 (8.35), 42–85	0.746
**Duration since Diagnosis (years)**	5.27 (3.70), 1–16	10.05 (4.90), 1–18	**<0.001**
**H&Y (1/2/3/4)**	0/27/3/0	0/16/6/0	0.209
**MDS-UPDRS-III (0–132)**	31.87 (12.29), 9–59	35.05 (14.32), 12–69	0.436
**LEDD (mg)**	556.83 (323.53), 0–1745	674.09 (392.07), 200–1715.45	0.259
**Mini-BESTest (0–28)**	23.53 (4.30), 9–28	22.73 (3.53), 14–28	0.168
**FAB-Scale (0–40)**	16.90 (1.18), 14–18	16.45 (2.04), 12–18	0.977
**MoCA (0–30)**	26.17 (2.21), 21–29	25.77 (2.93), 20–30	0.708
**TMT-A (s)**	48.36 (40.16), 21.74–239	41.69 (14.93), 25–85	0.409
**TMT-B (s)**	120.36 (82.25), 42–582	106.59 (47.37), 35–227	0.563
**FES-I (16–64)**	23.97 (8.86), 16–47	26.18 (9.49), 16–50	0.573
**NFOG-Q (1–28)**	-	15.37 (5.59), 6–26	-
**FOG-Score (0–36)**	0.633 (1.45), 0–6	4.18 (5.40) 0–18	**0.003**
**FOG ratio**	2.92 (2.81) 0.67–14.18	3.39 (4.12) 0.44–19.18	0.647

Abbreviations: Values represent mean (standard deviation), range, except for H&Y; MDS-UPDRS-III = Movement Disorder Society–Unified Parkinson’s Disease rating Scale Part III; H&Y = Hoehn and Yahr stage, LEDD = Levodopa equivalent daily dose; Mini-BESTest = Mini Balance Evaluation Systems Test; FAB-Scale = Fullerton Advanced Balance Scale, MoCA = Montreal Cognitive Assessment; TMT = Trail Making Test; FES-I = Falls Efficacy Scale-International; NFOG-Q = New Freezing of Gait Questionnaire.

### Correlations between APAs and first step-, gait- and FOG-related measures

At pretest, we found no significant correlations between APA size (AP and ML) and any of the other GI outcomes (Table 2). Regarding gait, we found significant moderate correlations of ML APA size with gait speed (p<0.001, r = 0.596) and stride length (p<0.001, r = 0.561) but only for the DT condition. Conversely, AP-APA size was not significantly correlated with gait speed or stride length. Table 2 shows the correlation results for the whole sample, and for freezers and non-freezers separately. Within Freezers only, our results showed neither ML APA size nor AP APA size was significantly correlated with any of the FOG-related outcomes (S1 Table).

**Table 2:** Correlations of APA measure with first step and gait measures at Pre.

APA measure	Other measure	Condition	All (n = 52)		Freezers (n = 22)		Non-freezers (n = 30)	
			correlation coefficient	p-value	correlation coefficient	p-value	correlation coefficient	p-value
**ML APA size**	**first step ROM**	ST	0.040	1.000	0.243	1.000	-0.209	1.000
		DT	0.194	0.692	0.317	0.603	0.054	1.000
**ML APA size**	**first step time**	ST	-0.244	0.350	-0.207	1.000	-0.418	0.107
		DT	-0.217	0.501	-0.008	1.000	-0.472	0.039*
**ML APA size**	**gait speed**	ST	0.294	0.152	0.507	0.065	0.013	1.000
		DT	0.596	<0.001**	0.608	0.011*	0.611	0.002**
**ML APA size**	**stride length**	ST	0.188	0.760	0.359	0.404	-0.081	1.000
		DT	0.561	<0.001**	0.639	0.005**	0.426	0.084
**AP APA size**	**first step ROM**	ST	0.048	1.000	0.432	0.179	-0.293	0.521
		DT	0.200	0.621	0.256	1.000	0.323	0.331
**AP APA size**	**first step time**	ST	-0.247	0.336	-0.417	0.215	-0.058	1.000
		DT	-0.140	1.000	-0.058	1.000	-0.437	0.071
**AP APA size**	**gait speed**	ST	-0.049	1.000	0.131	1.000	-0.200	1.000
		DT	0.051	1.000	0.041	1.000	0.199	1.000
**AP APA size**	**stride length**	ST	-0.080	1.000	0.0601	1.000	-0.218	1.000
		DT	0.071	1.000	0.111	1.000	0.054	1.000

Abbreviations: ML = medio-lateral, AP = anterior-posterior, APA = anticipatory postural adjustment, ROM = range of motion, ST = single task, DT = dual task.

### Effects of SBT- and regular treadmill training on GI

No significant time*training group interaction was found for any of the outcomes for GI-parameters. A significant time effect from Pre to Post was found for first step ROM (p = 0.003), APA Latency (p = 0.023) and APA duration (p = 0.032) under ST conditions, indicating larger first steps, quicker APAs and a shorter time to release the step after the training, irrespective of SBT or regular treadmill training. Additionally, no significant time*FOG-status interaction was detected, indicating that training effects were similar for freezers and non-freezers. For the overground gait measures, we also found significant time effects and no interaction effect. Participants were able to improve their gait speed (ST: p = 0.026; DT: p<0.001) and stride length (ST: p = 0.033; DT: p = 0.008) irrespective of the training mode. Further details with regard to the training effects are described in S2 Table. As there were no significant time*training-group interactions, the SBT- and the regular treadmill training groups were pooled for further correlation analysis.

### Correlation between training-induced changes in APAs and changes in first-step-, gait- and FOG-measures

**Fig 1:**
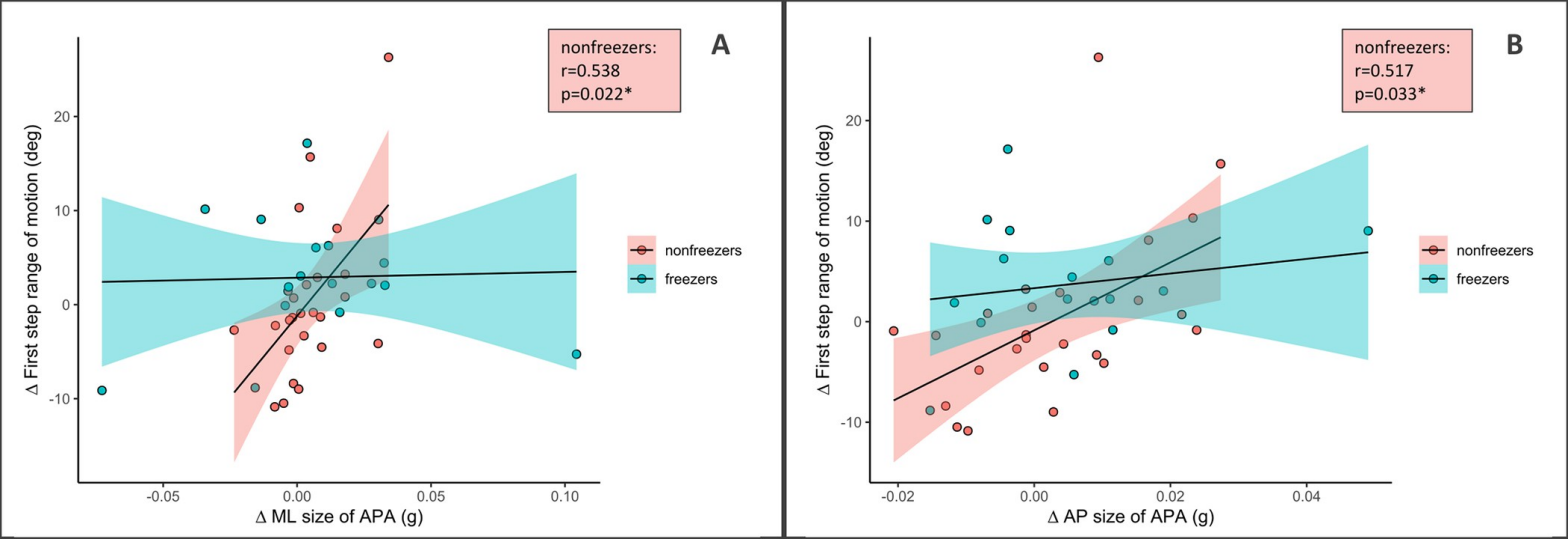
Association between training induced changes in (A) anterio-posterior (AP) and (B) medio-lateral (ML) size of APA and first step range of motion (ROM), respectively, for the dual task condition (DT).

The follow-up measurement was excluded in the analysis as there were not significant time effects present for the mixed model analysis. There were no significant correlations for the whole sample between the delta of APA size and the first step and gait outcomes. When analyzing the data of freezers and non-freezers separately (Table 3) there was a significant moderate correlation in the non-freezers for the delta of ML APA size and AP APA size with first step ROM during DT (p = 0.033, r = 0.517 and p = 0.022, r = 0.538, respectively) (Fig 1). This association remained significant when performing a regression analysis with disease duration as a covariate and delta of first step ROM as dependent variable and delta APA size as independent variable, respectively.

**Table 3:** Correlations of changes in APA measures with changes in first step and gait measures (Post-Pre).

APA measure	Other measure	Condition	All (n = 52)		Freezers (n = 22)		Non-freezers (n = 30)	
			correlation coefficient	p-value	correlation coefficient	p-value	correlation coefficient	p-value
**ML APA size Δ**	**first step ROM Δ**	ST	-0.114	1.000	-0.367	0.5906	0.322	0.620
		DT	0.191	0.907	0.033	1.000	0.517	0.033*
**ML APA size Δ**	**first step time Δ**	ST	-0.006	1.000	-0.071	1.000	0.082	1.000
		DT	-0.089	1.000	0.056	1.000	-0.314	0.505
**ML APA size Δ**	**gait speed Δ**	ST	0.066	1.000	0.296	0.9936	-0.137	1.000
		DT	0.292	0.242	0.422	0.3648	-0.069	1.000
**ML APA size Δ**	**stride length Δ**	ST	0.174	1.000	0.413	0.3983	-0.168	1.000
		DT	0.315	0.169	0.396	0.4617	-0.023	1.000
**AP APA size Δ**	**first step ROM Δ**	ST	-0.114	1.000	-0.124	1.000	-0.191	1.000
		DT	0.363	0.072	0.486	0.1915	0.538	0.022*
**AP APA size Δ**	**first step time Δ**	ST	0.020	1.000	-0.011	1.000	0.218	1.000
		DT	-0.014	1.000	0.025	1.000	-0.190	1.000
**AP APA size Δ**	**gait speed Δ**	ST	0.212	0.806	0.492	0.1799	0.093	1.000
		DT	0.190	0.913	0.384	0.5126	-0.375	0.259
**AP APA size Δ**	**stride length Δ**	ST	0.150	1.000	0.554	0.0837	-0.093	1.000
		DT	0.302	0.208	0.470	0.2281	-0.449	0.097

Abbreviations: ML = medio-lateral, AP = anterior-posterior, APA = anticipatory postural adjustment, ROM = range of motion, ST = single task, DT = dual task, Δ = delta (Post-Pre).

There were no significant correlations between the delta of ML and AP APA size with the delta of any of the gait or FOG-related measures.

## Discussion

The aim of this study was to investigate the meaning of APAs for the quality of locomotion in people with PD. We found an association between the ML size of the APA and gait speed as well as stride length during overground gait during the DT condition, indicating that those participants having larger ML APAs were those who walk faster with larger strides. The association being stronger during DT conditions suggests that APAs may reflect an automatic process, which is more relevant under DT condition. However, no such relationship was found for first step outcomes. Our findings are in line with previously reported results in healthy adults, who also showed larger ML APAs when walking at a faster speed [4]. However and in contrast to previous studies, we did not find this association for the AP direction [8]. There is only one previous study in PD on this topic, reporting an association of AP COP displacement with step length [33]. In healthy individuals, the AP APA is proposed to be predictive of motor performance while the ML APA is seen to be predictive of postural stability [34]. It is presently unclear whether this also applies to people with PD. Other work has shown that the effectiveness of an APA is not only determined by the APA amplitude but also COM velocity at toe-off plays an important role [35], which was not assessed in this study.

Regarding the association between APA outcomes and FOG-related measures we found no significant correlations between APAs and the FOG-related outcomes. In contrast, a recent study investigated the association between APA amplitude and FOG severity (FOG ratio) using linear multiple regression and found that these components were indeed negatively associated with each other [36]. However, in the study by Moreira-Neto et al. [36] participants only performed a GI simulating (leg raising in the lying position) task and did not actually perform GI, which could have led to different findings. In a study by Schlenstedt et al. it was reported that smaller ML APAs were associated with less subjective FOG [14], which we could not replicate in this sample. Generally, the presented findings provide evidence that APA size is unrelated to subjective and objective FOG at baseline.

We expected that SBT-training, requiring adapted weight shifts due to the changing belt speeds, would be superior to regular treadmill training for improving GI. However, the results did not support this hypothesis. For that reason, we pooled both training groups for further correlation analysis. We demonstrated both training types to be effective in improving first step ROM and inducing a quicker release of the first step as well as generating larger steps and a higher gait speed overall. These findings were observed in both Freezers and non-freezers. It is unclear whether these improvements are clinically meaningful. Hasegawa et al. (2019) found that first step ROM correlated with the motor part of the MDS-UPDRS, indicating larger ROM of the first step to be beneficial [37]. The average improvement of the size of AP-APA was 0.004g and for ML-APA 0.003g, which was not a significant gain. These enhancements were of slightly below significant changes induced by levodopa [20]. However, neither the gains found in this study nor the levodopa induced gains were larger than the minimal detectable change of 0.02g for AP-APA and 0.03g for ML-APA [38].

When correlating the training-related changes in the size of APA with changes in GI, gait and FOG-related measures, we found that larger AP- and ML-APAs improvements were associated with larger first steps ROM improvements during GI only in non-freezers and only during DT conditions. We could show that those differences were not driven by disease duration which was different in the subgroup of freezers and non-freezers. The lack of a significant correlation of the size of APA and first step ROM of our baseline data reduces the potential of a causal association between the generation of an APA and the size of the first step. Furthermore, there was no relationship between changes in the size of APAs and changes in any outcome of gait or FOG-related measures after training. We suspect there might be a potential ceiling effect for APA size under ST conditions. Our results are in line with a study of Amano et al. [23] who found improvements in APA size not being accompanied by increases in gait speed in people with PD. We speculate that a potential reason for those findings could be that the underlying mechanism for gait initiation and straight walking are distinct from each other to some extent and are modulated independently by the treadmill training. However, the presented data are insufficient to answer this question. Previous work in healthy individuals using functional near-infrared spectroscopy showed that the activation of the prefrontal cortex and the motor cortex was phase-dependent, supporting the hypothesis that the neural engagement differed between the preparation phase and steady-state walking [39]. However, this has not yet been investigated in PD. Only one study used fMRI while conducting a GI-simulating task in people with PD and showed that gait initiation did share neural correlates with gait automaticity (dual task cost on stride length), as they found an association with the mesencephalic locomotor region activation [36]. Those findings further support our speculation that APAs might be less relevant during ST gait and more relevant during DT conditions, as was shown with the presented results.

As for FOG, the findings presented here indicate that the mechanisms involved to generate the size of APAs seemed to be also less important for FOG. Furthermore, the limited associations in changes in APA and other outcomes were only found in non-freezers. This suggests that the role of APAs in gait initiation in freezers is different to that in non-freezers.

Considering that APAs are generally highly adaptable when the system is constrained biomechanically, physiologically or psychologically [40,41], this means that APAs are an outcome measure which is not always easy to interpret as not all factors can be controlled for. For example medication status in PD can have a great influence on the results and participants in this study were only tested ON medication. Furthermore, using a visual signal during GI assessment may have influenced the capturing of APAs, as giving an external start signal can act as a cue and improve GI [42]. Different outcome measures that could be interesting like first step trunk momentum were not assessed in this work, which adds to the limitations. Additionally, this work has the limitation that the training intervention was not specifically designed to improve gait initiation and did not reveal significant training effects on the size of APAs. However, we found large between-subject variation in our data, as indicated by large standard deviations, justifying the present exploratory correlation analysis.

## Conclusions

The findings of this work revealed new insights into how APAs were not associated with first step characteristics and freezing-related measures in people with PD. Yet, baseline APAs were found to relate with dual task gait characteristics, indicating that APA size might be less relevant during gait alone and more relevant when cognitive resources are divided by introducing an additional cognitive task. We have also shown that SBT and regular treadmill training were similarly effective to improve GI and gait. Training-induced changes in the size of APAs were related to training benefits in the first step ROM only in non-freezers, suggesting a different role of APAs in the freezers. No associations were found between changes in APAs and gait or freezing-related measures which suggests that the training-related improvements in overall gait quality (gait speed and stride length) could be achieved without necessarily increasing APA size. Overall, our findings suggest that increasing APA size through interventions might not be the ideal target for overall improvement of gait initiation and gait.

## Supporting information

S1 Fig Flow diagram of recruitment process and study conduction. n = number of participants; CAU = Christian-Albrechts-University Kiel, KUL = KU Leuven, SBT = Split-Belt treadmill, TBT = Tied-Belt treadmill, *intention-to-treat based analysis.
(TIF)

S1 Table Correlations of APA measures and FOG-related measures at Pre in Freezers. ML = medio-lateral, AP = anterior-posterior, APA = anticipatory postural adjustment, ROM = range of motion, FOG = Freezing of Gait, GI = gait initiation, NFOGQ = New Freezing of Gait Questionnaire, † Pearson correlation was used for interval-scaled variables (FOG ratio), Spearman was used for ordinal scaled variables (FOG score, FOG score (GI), NFGOQ, NFOGQ (GI), % time frozen).
(DOCX)

S2 Table Descriptive values and Linear Mixed model results for APA, first step and gait outcomes. SBT = Split-Belt treadmill, TBT = Tied-Belt, ST = single task, DT = dual task, ML = medio-lateral, AP = anterior-posterior, APA = anticipatory postural adjustment, ROM = range of motion, † p-values of linear mixed models and post-hoc tests are based on log-transformed variables due to non-normality of residuals. Additionally testing for baseline differences was done using non-parametric methods (man-Whitney-U-Test) in the indicated variables.
(DOCX)
