# Supplementary figures and images for: Towards a better understanding of anticipatory postural adjustments in people with Parkinson’s disease
Source: PLoS One. 2024 Mar 11;19(3):e0300465. doi: 10.1371/journal.pone.0300465 (PMC10927092; doi:10.1371/journal.pone.0300465)

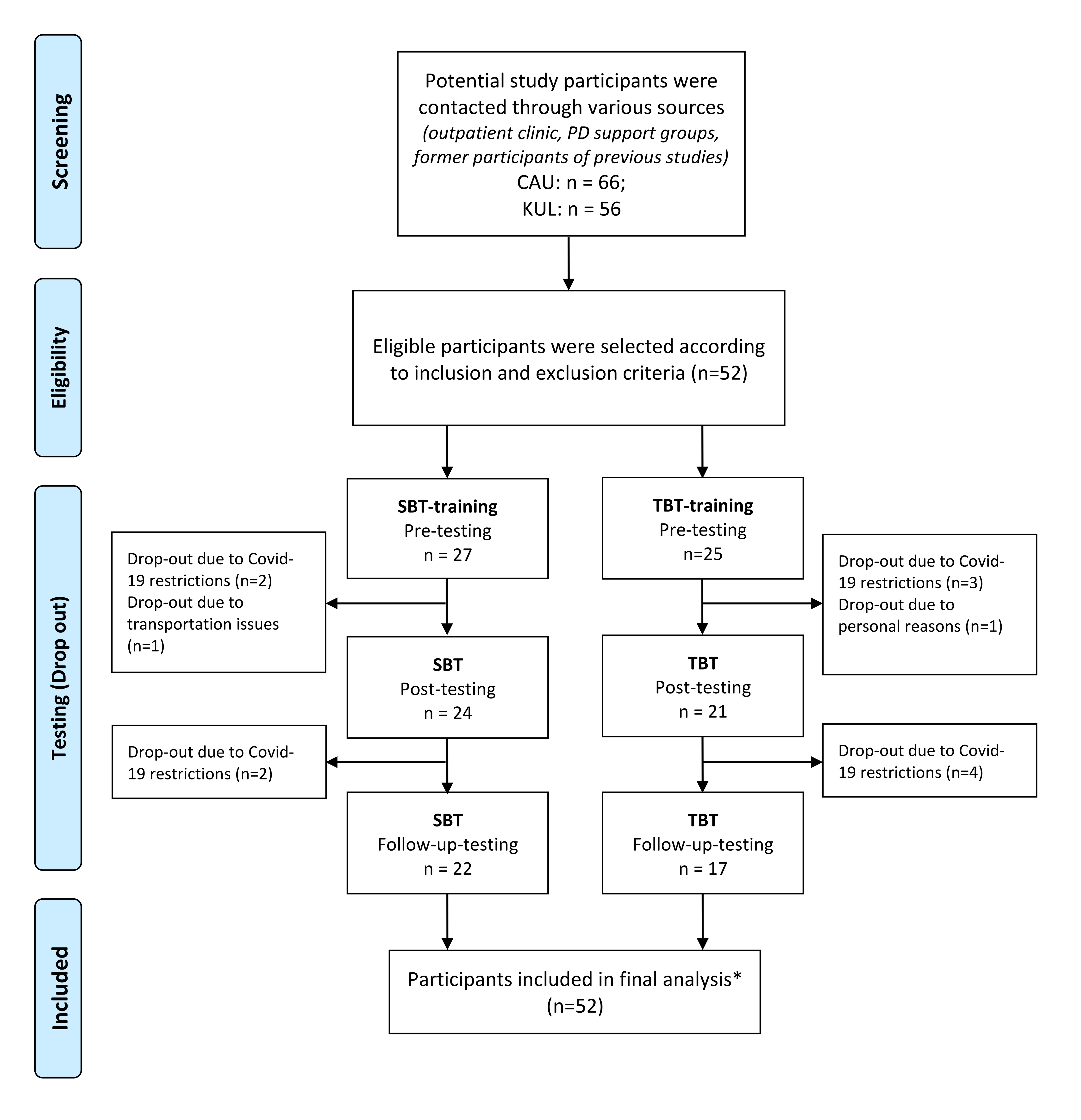

Supplement: S1 Fig — n = number of participants; CAU = Christian-Albrechts-University Kiel, KUL = KU Leuven, SBT = Split-Belt treadmill, TBT = Tied-Belt treadmill, *intention-to-treat based analysis. (TIF) [file pone.0300465.s001.tif]
