# Supplementary material for: Towards a better understanding of anticipatory postural adjustments in people with Parkinson’s disease
Source: PLoS One. 2024 Mar 11;19(3):e0300465. doi: 10.1371/journal.pone.0300465 (PMC10927092; doi:10.1371/journal.pone.0300465)
Supplement: S1 Table — ML = medio-lateral, AP = anterior-posterior, APA = anticipatory postural adjustment, ROM = range of motion, FOG = Freezing of Gait, GI = gait initiation, NFOGQ = New Freezing of Gait Questionnaire, † Pearson correlation was used for interval-scaled variables (FOG ratio), Spearman was used for ordinal scaled variables (FOG score, FOG score (GI), NFGOQ, NFOGQ (GI), % time frozen). (DOCX) [file pone.0300465.s002.docx]

Supplementary Table 1 Correlations of APA measures and FOG-related measures at Pre in Freezers

| **APA measure** | **FOG measures** | **condition** | **correlation coefficient†** | **p-value** |
| --- | --- | --- | --- | --- |
| **ML APA size** | **FOG ratio** | ST | -0.091 | 1.000 |
|  |  | DT | -0.446 | 0.188 |
| **ML APA size** | **FOG score** | ST | -0.280 | 0.612 |
|  |  | DT | -0.322 | 0.603 |
| **ML APA size** | **FOG score (GI)** | ST | -0.505 | 0.174 |
|  |  | DT | -0.442 | 0.403 |
| **ML APA size** | **NFOGQ** | ST | 0.089 | 1.000 |
|  |  | DT | -0.253 | 1.000 |
| **ML APA size** | **NFOGQ (GI)** | ST | 0.144 | 1.000 |
|  |  | DT | -0.126 | 1.000 |
| **ML APA size** | **% time frozen** | ST | -0.399 | 0.910 |
|  |  | DT | -0.534 | 0.437 |
| **AP APA size** | **FOG ratio** | ST | -0.096 | 1.000 |
|  |  | DT | -0.138 | 1.000 |
| **AP APA size** | **FOG score** | ST | -0.047 | 1.000 |
|  |  | DT | -0.026 | 1.000 |
| **AP APA size** | **FOG score (GI)** | ST | 0.138 | 1.000 |
|  |  | DT | -0.146 | 1.000 |
| **AP APA size** | **NFOGQ** | ST | 0.592 | 0.630 |
|  |  | DT | 0.315 | 1.000 |
| **AP APA size** | **NFOGQ (GI)** | ST | 0.442 | 0.780 |
|  |  | DT | 0.443 | 1.000 |
| **AP APA size** | **% time frozen** | ST | -0.121 | 1.000 |
|  |  | DT | -0.151 | 1.000 |

ML=medio-lateral, AP=anterior-posterior, APA=anticipatory postural adjustment, ROM= range of motion, FOG=Freezing of Gait, GI=gait initiation, NFOGQ=New Freezing of Gait Questionnaire

† Pearson correlation was used for interval-scaled variables (FOG ratio), Spearman was used for ordinal scaled variables (FOG score, FOG score (GI), NFGOQ, NFOGQ (GI), % time frozen)
