# Supplementary material for: Towards a better understanding of anticipatory postural adjustments in people with Parkinson’s disease
Source: PLoS One. 2024 Mar 11;19(3):e0300465. doi: 10.1371/journal.pone.0300465 (PMC10927092; doi:10.1371/journal.pone.0300465)
Supplement: S2 Table — SBT = Split-Belt treadmill, TBT = Tied-Belt, ST = single task, DT = dual task, ML = medio-lateral, AP = anterior-posterior, APA = anticipatory postural adjustment, ROM = range of motion, † p-values of linear mixed models and post-hoc tests are based on log-transformed variables due to non-normality of residuals. Additionally testing for baseline differences was done using non-parametric methods (man-Whitney-U-Test) in the indicated variables. (DOCX) [file pone.0300465.s003.docx]

Supplementary Table 2 Descriptive values and Linear Mixed model results for APA, first step and gait outcomes

| **Outcome** | **Time** | **SBT (n=27)** | | | **TBT (n=25)** | | | **Effect size** | **Between group comparison at Pre**  **(*p*-value)** | **Between group comparison of differences (*p*-value)** | **Time-effect / group*time interaction**  **(*p*-values)** |
| --- | --- | --- | --- | --- | --- | --- | --- | --- | --- | --- | --- |
|  |  | **Mean (SD)** | **Median**  **(Q1-Q3)** | **Post hoc comparison (*p*-value)** | **Mean (SD)** | **Median**  **(Q1-Q3)** | **Post hoc comparison (*p*-value)** |  |  |  |  |
| **APA outcomes** | | | | | | | | | | | |
| **ML APA size (g)**  **(ST)** | Pre | 0.041 (0.019) | 0.040 (0.031-0.046) |  | 0.038 (0.018) | 0.036  (0.024-0.051) |  |  | 0.705 |  | 0.215/0.644 |
|  | Pre-Post | 0.005 (0.017) | 0.001 (-0.005-0.014) | 0.471 | 0.003 (0.020) | -0.004  (-0.015-0.020) | 0.978 | 0.127 |  | 0.464 |  |
|  | Pre-Follow-up | 0.002 (0.019) | 0.002 (-0.012-0.011) | 0.951 | 0.002 (0.015 | -0.001  (-0.006-0.010) | 0.970 | 0.0471 |  | 0.995 |  |
| **ML APA size (g) (DT)** | Pre | 0.041 (0.020) | 0.039 (0.027-0.048) |  | 0.038 (0.017) | 0.039  (0.025-0.051) |  |  | 0.269 |  | 0.202/0.692 |
|  | Pre-Post | 0.008 (0.029) | 0.005 (-0.002-0.015) | 0.576 | 0.003 (0.017) | 0.002  (-0.003-0.013) | 0.981 | 0.118 |  | 0.543 |  |
|  | Pre- Follow-up | 0.002 (0.026) | 0.002 (-0.009-0.010) | 0.999 | 0.003 (0.020) | 0.002  (-0.007-0.010) | 0.999 | 0.044 |  | 0.958 |  |
| **AP APA size (g) (ST)** | Pre | 0.040 (0.016) | 0.039 (0.029-0.050) |  | 0.042 (0.017) | 0.036  (0.033-0.046) |  |  | 0.785 |  | 0.243/0.758 |
|  | Pre-Post | 0.004 (0.017) | 0.006 (-0.007-0.014) | 0.533 | 0.003 (0.019) | 0.004  (-0.001-0.011) | 0.984 | 0.035 |  | 0.465 |  |
|  | Pre- Follow-up | 0.002 (0.025) | 0.001 (-0.019-0.019) | 0.963 | 0.000 (0.016) | 0.003  (-0.010-0.010) | 0.999 | 0.089 |  | 0.725 |  |
| **AP APA size (g) (DT)** | Pre | 0.046 (0.041) | 0.037 (0.028-0.050) |  | 0.035 (0.017) | 0.034  (0.027-0.038) |  |  | 0.076 |  | 0.916/0.564 |
|  | Pre-Post | -0.003 (0.042) | 0.005 (-0.011-0.011) | 0.999 | 0.003 (0.009) | 0.001  (-0.004-0.009) | 1.000 | 0.025 |  | 0.840 |  |
|  | Pre- Follow-up | -0.005 (0.046) | 0.004 (-0.009-0.015) | 0.972 | 0.004 (0.010) | 0.003  (-0.001-.008) | 0.999 | 0.637 |  | 0.499 |  |
| **APA duration (s) (ST)†** | Pre | 0.607 (0.284) | 0.577 (0.388-0.794) |  | 0.640 (0.284) | 0.588  (0.528-0.667) |  |  | 0.087 |  | 0.032*/0.788 |
|  | Pre-Post | -0.108 (0.339) | -0.022 (-0.344-0.049) | 0.305 | -0.038 (0.191) | -0.005  (-0.166-0.087) | 0.524 | 0.170 |  | 0.776 |  |
|  | Pre- Follow-up | -0.048 (0.395) | -0.087 (-0.179-0.119) | 0.609 | 0.028 (0.268) | -0.068  (-0.169-0.201) | 0.996 | 0.187 |  | 0.495 |  |
| **APA duration (s) (DT)†** | Pre | 0.544 (0.196) | 0.531 (0.400-0.665) |  | 0.581 (0.205) | 0.560  (0.445-0.662) |  |  | 0.535 |  | 0.937/0.186 |
|  | Pre-Post | -0.025 (0.261) | -0.041 (-0.107-0.034) | 0.976 | 0.122 (0.273) | 0.047  (0.019-0.254) | 0.758 | 0.198 |  | 0.112 |  |
|  | Pre- Follow-up | 0.043 (0.304) | -0.030 (-0.136-0.256) | 1.000 | 0.030 (0.215) | 0.051  (-0.046-0.180) | 0.997 | 0.218 |  | 0.389 |  |
| **First step outcomes** | | | | | | | | | | | |
| **APA latency (s)**  **(ST)†** | Pre | 0.775 (0.324) | 0.706 (0.589-0.914) |  | 0.767 (0.274) | 0.734  (0.634-0.802) |  |  | 0.248 |  | 0.023*/0.440 |
|  | Pre-Post | -0.155 (0.365) | -0.118 (-0.439- -0.006) | 0.161 | -0.035 (0.168) | 0.009  (-0.122-0.093) | 0.912 | 0.276 |  | 0.253 |  |
|  | Pre- Follow-up | -0.046 (0.383) | -0.081 (-0.188-0.049) | 0.813 | 0.027 (0.236) | -0.038  (-0.144-0.190) | 1.000 | 0.168 |  | 0.348 |  |
| **APA latency (s) (DT)†** | Pre | 0.687 (0.218) | 0.625 (0.539-0.834) |  | 0.743 (0.308) | 0.682  (0.578-0.768) |  |  | 0.843 |  | 0.323/0.182 |
|  | Pre-Post | -0.004 (0.286) | -0.016 (-0.126-0.123) | 0.999 | 0.144 (0.386) | 0.078  (-0.023-0.300) | 0.502 | 0.307 |  | 0.072 |  |
|  | Pre- Follow-up | 0.044 (0.266) | -0.010 (-0.131-0.143) | 1.000 | -0.021 (0.345) | 0.027  (-0.030-0.184) | 1.000 | 0.187 |  | 0.655 |  |
| **First step ROM (°) (ST)** | Pre | 29.662 (9.209) | 28.888 (24.445-36.034) |  | 32.381 (9.614) | 30.456  (24.746-39.501) |  |  | 0.003* |  | 0.003*/0.258 |
|  | Pre-Post | 2.154 (6.441) | 1.647 (-0.097-3.560) | 0.646 | 4.672 (5.151) | 3.506  (1.731-7.608) | 0.012* | -0.267 |  | 0.148 |  |
|  | Pre- Follow-up | 1.968 (7.901) | 0.215 (-4.168-8.581) | 0.791 | 1.335 (6.964) | -0.314  (-3.437-4.816) | 0.912 | 0.067 |  | 0.928 |  |
| **First step ROM (°) (DT)** | Pre | 27.756 (8.355) | 28.935 (21.994-31.409) |  | 30.454 (10.042) | 29.901  (21.593-38.068) |  |  | 0.060 |  | 0.353/0.083 |
|  | Pre-Post | -0.636 (6.776) | -0.835 (-4.895-2.251) | 0.999 | 3.295 (8.125) | 1.999  (-2.345-6.727) | 0.198 | 0.252 |  | 0.055 |  |
|  | Pre- Follow-up | 1.114 (10.279) | 2.242 (-4.653-7.370) | 0.979 | 0.434 (3.918) | 1.567  (-2.549-2.579) | 0.999 | 0.063 |  | 0.815 |  |
| **First step time (s) (ST)†** | Pre | 0.448 (0.238) | 0.376 (0.334-0.428) |  | 0.370 (0.095) | 0.366  (0.309-0.423) |  |  | 0.044* |  | 0.262/0.057 |
|  | Pre-Post | -0.089 (0.246) | -0.031 (-0.108-0.018) | 0.201 | -0.006 (0.084) | -0.003  (-0.060-0.031) | 1.000 | 0.335 |  | 0.056 |  |
|  | Pre- Follow-up | -0.043 (0.101) | -0.039 (-0.058- -0.006) | 0.097 | 0.008 (0.107) | 0.028  (-0.061-0.056) | 0.936 | 0.209 |  | 0.025* |  |
| **First step time (s) (DT)†** | Pre | 0.393 (0.141) | 0.395 (0.293-0.438) |  | 0.420 (0.194) | 0.367  (0.320-0.473) |  |  | 0.379 |  | 0.524/0.895 |
|  | Pre-Post | -0.011 (0.130) | -0.023 (-0.059-0.036) | 0.999 | 0.000 (0.221) | 0.012  (-0.071-0.104) | 0.999 | 0.386 |  | 0.642 |  |
|  | Pre- Follow-up | -0.027 (0.127) | -0.023 (-0.062-0.042) | 0.990 | -0.039 (0.176) | -0.004  (-0.038-0.037) | 0.999 | 0.241 |  | 0.842 |  |
| **Overground gait outcomes** | | | | | | | | | | | |
| **Gait speed (m/s) (ST)** | Pre | 1.223(0.212) | 1.203(1.087-1.379) | - | 1.256 (0.206) | 1.308  (1.167-1.371) | - | - | 0.564 |  | 0.026*/0.143 |
|  | Pre-Post | 0.077(0.152) | 0.040(-0.022-0.185) | 0.004** | 0.010(0.070) | -0.025  (-0.038-0.033) | 0.729 | 0.322 | - | 0.071 |  |
|  | Pre- Follow-up | 0.095(0.158) | 0.057(0.009-0.208) | <0.001*** | 0.025(0.089) | 0.007  (-0.030-0.079) | 0.584 | 0.330 | - | 0.050 |  |
| **Gait speed (m/s) (DT)** | Pre | 1.132(0.256) | 1.056(0.971-1.327) | - | 1.130(0.264) | 1.201  (0.946-1.301) | - | - | 0.978 |  | <0.001*/0.154 |
|  | Pre-Post | 0.092(0.130) | 0.084(-0.0010-0.144) | <0.001*** | 0.029(0.073) | 0.019  (-0.013-0.074) | 0.217 | 0.241 | - | 0.057 |  |
|  | Pre- Follow-up | 0.093(0.128) | 0.075(0.001-0.198) | <0.001*** | 0.049(0.098) | 0.030(-0.023-0.116) | 0.096 | 0.171 | - | 0.098 |  |
| **Stride length (mm) (ST)** | Pre | 1316.099 (176.139) | 1332.799 (1240.615-1447.125) | - | 1345.004 (191.348) | 1392.589 (1252.933-1474.218) | - | - | 0.574 | - | 0.033*/0.103 |
|  | Pre-Post | 49.627 (101.509) | 42.206 (-3.710-92.177) | 0.050 | 16.679 (46.326) | -2.554 (-20.847-51.847) | 0.943 | 0.179 | - | 0.175 |  |
|  | Pre- Follow-up | 65.247 (122.804) | 55.979 (4.023-120.9010) | 0.021 | 11.231 (56.980) | -4.979 (-22.288-52.137) | 0.999 | 0.290 | - | 0.0432 |  |
| **Stride length (mm) (DT)** | Pre | 1228.169 (215.242) | 1234.263 (1087.263-1371.316) | - | 1246.751 (226.839) | 1302.764 (1101.350-1391.440) | - | - | 0.764 | - | 0.008*/0.275 |
|  | Pre-Post | 65.133 (111.285) | 55.514 (0.843-105.930) | 0.015 | 19.108 (64.764) | 17.392 (-18.127-54.621) | 0.901 | 0.208 | - | 0.116 |  |
|  | Pre- Follow-up | 59.584 (120.390) | 54.306 (0.806-106.065) | 0.017 | 28.978 (69.333) | 24.514  (-20.863-92.241) | 0.857 | 0.138 | - | 0.151 |  |

† p-values of linear mixed models and post-hoc tests are based on log-transformed variables due to non-normality of residuals. Additionally testing for baseline differences was done using non-parametric methods (man-Whitney-U-Test) in the indicated variables.
